# Lateral habenula deep brain stimulation for personalized treatment of drug addiction

**DOI:** 10.3389/fnhum.2013.00806

**Published:** 2013-12-12

**Authors:** Gal Yadid, Iris Gispan, Elad Lax

**Affiliations:** ^1^The Neuropsychopharmacology Lab, The Mina and Everard Goodman Faculty of Life Sciences, Bar-Ilan UniversityRamat-Gan, Israel; ^2^The Leslie and Susan Gonda (Goldschmied) Multidisciplinary Brain Research Center, Bar-Ilan UniversityRamat-Gan, Israel

**Keywords:** limbic system, negative reward, cocaine, diffusion tensor imaging, fasciculus retroflexus

Drug addiction is a major brain disease, and a serious clinical and social problem. The number of adults who require substance abuse treatment is anticipated to escalate from 1.7 million in 2000 and 2001 to 4.4 million in 2020 (Gfroerer et al., 2003). Addiction is a gradual process, which begins with occasional use, proceeds to regular use and finally progresses to uncontrollable abuse. The main problem is the high rates of relapse among abusers who have ceased drug use. Cocaine, in particular, is one of the most prevalent recreational drugs, with especially high relapse rates (Bossert et al., 2005). To date, there are no approved pharmacological treatments for stimulant drugs of abuse such as cocaine.

A new class of neurosurgical interventions is currently being developed and used for the treatment of movement disorders and disorders of mood and behavior (Mathews, 2011). One of the prominent treatments is deep brain stimulation (DBS), in which implanted electrodes deliver electrical stimulation to stereotactically targeted brain regions. DBS in selected brain regions has shown significant therapeutic benefits for otherwise treatment-resistant movement disorders, including Parkinson's disease, essential tremor and Dystonia (Kringelbach et al., 2007). The main reasons of the success of this method are its reversibility (as opposed to lessoning techniques), adaptability, controlled usage, and low morbidity (Benabid and Torres, 2012). Thus, DBS research has been extended to various brain regions for treatment of neuropsychiatric conditions such as Alzheimer's disease, Tourette's syndrome, obsessive-compulsive disorder and depression (Krack et al., 2010; Mathews, 2011). Recent research in both animals and humans has indicated that DBS may also be an effective treatment for addiction. DBS was tested for its effect on response to alcohol, cocaine, heroin, morphine and nicotine, showing promising results in several regions of the reward system. The nucleus accumbens, which receives dopaminergic input from the ventral tegmental area (VTA) and plays a key role in cocaine addiction, was suggested as a primary target for DBS (Luigjes et al., 2012). However, we postulate that better results may be obtained by targeting more remote limbic regions which regulate the mesolimbic dopaminergic system, such as the lateral habenula (LHb).

The LHb is a dorsal diencephalic structure located lateral to the third ventricle. This region receives inputs from several parts of the limbic system, including the bed nucleus of stria terminalis, lateral preoptic area, lateral hypothalamus and nucleus accumbens, among others (Lecourtier and Kelly, 2007). Another major source of inputs to the LHb is the internal globus pallidus (GPi) (Hikosaka et al., 2008). LHb efferents project mainly through the fasciculus retroflexus (FR) to several midbrain nuclei including the raphe nucleus, rostro-medial tegmentum (RMTg), ventral-tegmental area (VTA), substantia nigra and locus coeruleus.

**Figure 1:**
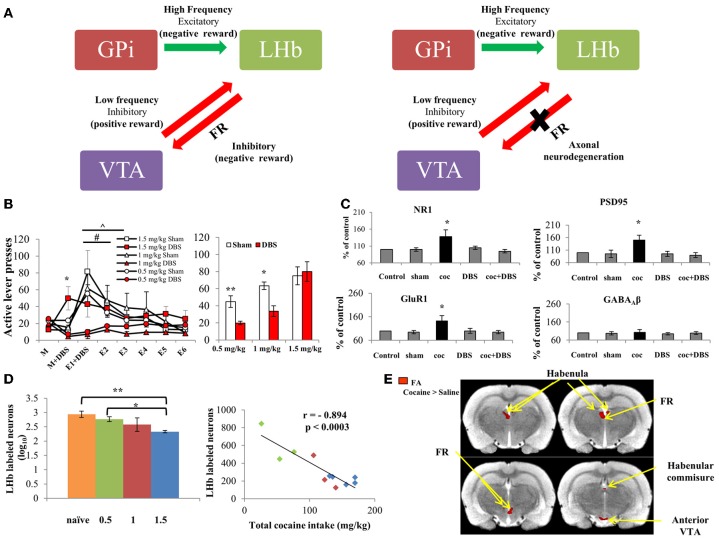
**(A)** Scheme representing the main LHb afferents and efferents. Left: The LHb receives excitatory inputs from the GPi, which encode for aversion. The LHb subsequently fires to inhibit the VTA, which is the main dopaminergic source that activates the reward system. Thus, the GPi-LHb pathway creates negative reward signals. In contrast, when a positive reward is predicted or presented, the VTA activates the reward system and also inhibits its regulator, the LHb, thus encoding for positive reward signal. Right: Repeated intake of cocaine dose-dependently degenerate the main LHb-to-VTA fibers, which comprise the FR. This ceases LHb regulation of the VTA, which consequently renders LHb DBS ineffective at high doses (below). **(B)** Left: Effects of LHb DBS on cocaine-seeking behaviors. Rats were allowed to self-administer cocaine (FR-1 schedule; 0.5, 1 or 1.5 mg/kg; *n* = 15, 6, and 9, respectively). After reaching stable maintenance levels (day M), rats from each group were divided and either treated with DBS or sham (day M + DBS). 0.5 mg/kg and 1 mg/kg cocaine-exposed groups treated with DBS showed decreased active lever presses compared to the DBS-treated 1.5 mg/kg group (^*^p < 0.001 for both). DBS treatment was given again during the first extinction session, and extinction responding was measured for 6 days (E1 + DBS through E6). A significantly accelerated rate of extinction was found for the 0.5 and 1 mg/kg DBS-treated groups compared to respective controls (^∧^, # *p* < 0.001). No changes were found between DBS-treated and sham operated rats trained to self-administer 1.5 mg/kg cocaine. Right: Effect of LHb DBS on reinstatement. After E6, rats were reinstated to cocaine by a priming cocaine injection (10 mg/kg, i.p.) and light-cue. Rats which received DBS treatment on E1 and which self-administered either 0.5 or 1 mg/kg cocaine showed significantly reduced active lever presses compared to controls (^**^p < 0.005 and ^*^p < 0.05, respectively). However, no differences were found between DBS-treated and sham-operated rats trained for 1.5 mg/kg cocaine. Values are expressed as mean ± SEM, in both graphs. **(C)** Effect of DBS of the LHb on levels of NR1, GluR1 and PSD95 in the VTA. Protein levels of the NR1 subunit of the NMDA receptor, GluR1 subunit of the AMPA receptor and scaffolding protein PSD95 were increased following cocaine self-administration (FR-1 schedule, 0.5 mg/kg). DBS of the LHb in cocaine-trained rats restored NR1, GluR1 and PSD95 levels to normal. This effect was specific to the glutamatergic system, since levels of the GABA_A_ receptor β subunits (β2 and β3) remained unchanged. DBS alone did not alter levels of these proteins in the VTA (values are expressed as mean ± SEM). ^*^p < 0.001 for cocaine-treated rats (coc) vs. control, sham-operated, DBS-treated and cocaine + DBS-treated rats (coc+DBS). **(D)** Cocaine-induced FR neurodegeneration. Left: Labeled LHb neurons in cocaine-treated vs. naïve rats. One day after reaching stable maintenance levels in the self-administration paradigm (FR-1 schedule; 0.5, 1 or 1.5 mg/kg; *n* = 3–5 per group), the amount of fluorogold-labeled LHb neurons was significantly reduced in 1.5 mg/kg cocaine-treated rats as compared to 0.5 mg/kg cocaine-treated (^*^p < 0.002) and naïve rats (^**^p < 0.001), indicating considerable neurodegeneration of the LHb-midbrain circuit at high dose cocaine. Values are expressed as mean ± SEM. Right: Correlation between total cocaine intake and labeled LHb neurons. A substantial, reverse correlation was found between the amount of labeled LHb neurons and total cocaine intake (mg/kg) (*r* = −0.894; *p* < 0.0003). **(E)** Statistical parametric maps of FA values for cocaine- vs. saline-treated rats. Rats were trained to self-administer cocaine (1.5 mg/kg, *n* = 6) or saline (*n* = 5) for 11 days. MRI followed by DTI analysis showed a significant increase in FA values in all regions of interest in rats which self-administered 1.5 mg/kg cocaine, compared to controls. No difference in FA values was found in the ventral posterior thalamus and the substantia nigra (served as control ROI; Lax et al., 2013). With permission from Friedman et al. (2010); Lax et al. (2013).

Excitatory innervations from the GPi send reward-related signals encoding for aversion, thus regulating LHb activity (Hong and Hikosaka, 2008; Shabel et al., 2012). When the LHb is activated, it controls dopaminergic midbrain neurons both directly (Brinschwitz et al., 2010) and indirectly, via a bi-synaptic connection through the RMTg (Jhou et al., 2009a,b; Omelchenko et al., 2009; Balcita-Pedicino et al., 2011), leading to almost complete inhibition of all dopaminergic neurons (Ji and Shepard, 2007). This reduces dopaminergic cell firing, consequently lowering motivation and reward (Matsumoto and Hikosaka, 2007) (Figure 1A). Given the pivotal role of the LHb in regulation of midbrain nuclei activity and therefore in reward-related behaviors, it was suggested that modulation of this region by DBS might be an effective therapeutic tool for psychiatric disorders, including major depression (Sartorius and Henn, 2007; Hauptman et al., 2008), and drug addiction (Luigjes et al., 2012). This hypothesis was strengthened by high-resolution MRI studies in humans, demonstrating reduced LHb volume in bipolar disorder and major depressive disorder (Savitz et al., 2011). In addition, a new study in rat models of depression revealed that tetanic, high-frequency DBS of the LHb suppressed synaptic activity of LHb VTA-projecting neurons and improved depressive-like behaviors (Li et al., 2011). This effect was similar to, though more potent than, results of pharmacological inhibition (Winter et al., 2011) and lesion of the LHb (Yang et al., 2008). Moreover, application of DBS to the LHB of two patients with treatment-resistant depression demonstrated promising outcomes (Sartorius et al., 2010; Kiening and Sartorius, 2013). These results raise the possibility that LHb DBS, and especially high-frequency stimulation, causes a transient “functional lesion” that reduces LHb inhibition of midbrain nuclei.

Accumulating evidence shows involvement of the LHb in the addiction process, and recent findings also strongly imply that application of DBS in the LHb may serve as a potential treatment for drug addiction. Increased LHb activation is associated with exposure to several drugs of abuse, including experimenter-delivered cocaine, cocaine-associated cues (Brown et al., 1992, 2010; Franklin and Druhan, 2000) and heroin-associated cues (Zhang et al., 2005) (but see: Martin et al., 1997). This elevation in LHb activity may represent a form of homeostatic regulation on the reward system: excitatory LHb activity counteracts the robust increase in dopaminergic tone following cocaine exposure by inhibiting midbrain dopaminergic neurons, which consequently diminishes the drug's rewarding effect (Jhou et al., 2013; Zuo et al., 2013).

In a recent study in rats, we combined LHb stimulation frequencies. We found that a combination consisting of sequential high and low frequencies attenuated cocaine self-administration, extinction response, and drug- and cue-induced reinstatement (Figure 1B). Moreover, combined frequency DBS of the LHb normalized cocaine-induced increases in NMDA and AMPA receptor subunits and in scaffold protein PSD-95 (Figure 1C) (Friedman et al., 2010). It is notable that combined DBS of the LHB seems to be context-dependent, effectively reduced cocaine and sucrose self-administration (Friedman et al., 2010, 2011).

How does the combination of low and high stimulation patterns effectively reduce cocaine-seeking behavior? It was shown that high frequency excitatory inputs from the GPi activate the LHb following aversive or frustrating experience (Shabel et al., 2012). Conversely, low frequency inputs from other brain circuits, including the VTA dopaminergic system, inhibit LHb activity following positive reward expectation (Fiorillo et al., 2003). Thus, we propose that the combination of low- and high- stimulation patterns may mimic these different LHb inputs, resembling a state in which a known reward (in this case, cocaine) is repeatedly expected due to introduction of low frequency DBS, and each expectation event is immediately eliminated due to prompt presentation of high frequency DBS. Thus, application of combined stimulation hastens extinction learning.

Prolonged excitation of LHb neurons due to continuous administration of various drugs, including cocaine, d-amphetamine, methamphetamine (Lipton et al., 1991; Ellison et al., 1996; Meshul et al., 1998), cathinone, MDMA and nicotine (Carlson et al., 2000), eventually induces LHb neurotoxicity followed by substantial neurodegeneration of the habenula efferent fiber, i.e., the FR (Figure 1A). Moreover, continuous, intensive cocaine administration is associated with a long-lasting decrease in GABAergic synaptic density in rat LHb (Meshul et al., 1998). This may lead to increased excitatory activity of LHb neurons, which encode negative reward. Recent findings show that the efficacy of combined DBS of the LHb is reduced following intense, high-dose cocaine (1.5 mg/kg) self-administration in rats, as opposed to the treatment's beneficial effect on lower doses (0.5 and 1 mg/kg) at maintenance, extinction and reinstatement stages (Lax et al., 2013). The decreased efficacy of LHb DBS at high-dose cocaine is probably due to considerable FR degeneration (Figure 1D) (Lax et al., 2013), which decreases LHb inhibition of midbrain neurons. Nevertheless, LHb DBS as a treatment for severe cocaine abuse has considerable therapeutic potential, as shown by recent findings. Non-invasive MRI imaging followed by tensor diffusion imaging (DTI) analysis, which reveals abnormalities in white matter fiber structure, was used to detect alterations in the habenula-midbrain circuitry. Analysis demonstrated elevated fractional anisotropy and axial diffusivity in several parts of the habenula-midbrain circuit, including the LHb, FR, VTA, and habenular commissure of rats that self-administered high-dose cocaine (Lax et al., 2013) (Figure 1E). Thus, usage of DTI prior to DBS application may be valuable as a preoperative, personalized evaluation tool. DTI biomarkers can assist in determining the prospects for heavy addicts to benefit from LHb DBS treatment, consequently increasing positive outcome.

In summary, the LHb is emerging as a prominent target site for DBS treatment of cocaine addiction. However, abnormal brain connectivity following excessive cocaine exposure may result in inferior treatment outcomes. As in many other therapies, treatment of addiction also exhibits a wide variability in longitudinal efficacy. Therefore, early identification of factors which reduce treatment efficacy can assist in establishing inclusion and exclusion criteria, and facilitate optimal patient management. This supports the use of brain imaging for monitoring cocaine-induced alterations in brain anatomy and fiber connectivity, prior to DBS treatment. Specifically, DTI biomarkers for detection of cocaine-induced alterations in FR anatomy may be useful for identification and selection of potential responders to LHb DBS. Therefore, LHb electrical stimulation, with DTI as a non-invasive, pre-surgical diagnostic tool, may serve as an individualized treatment for drug addiction disorders, mainly for cases in which more conventional treatments such as psychotherapy and pharmacological treatments have failed.
